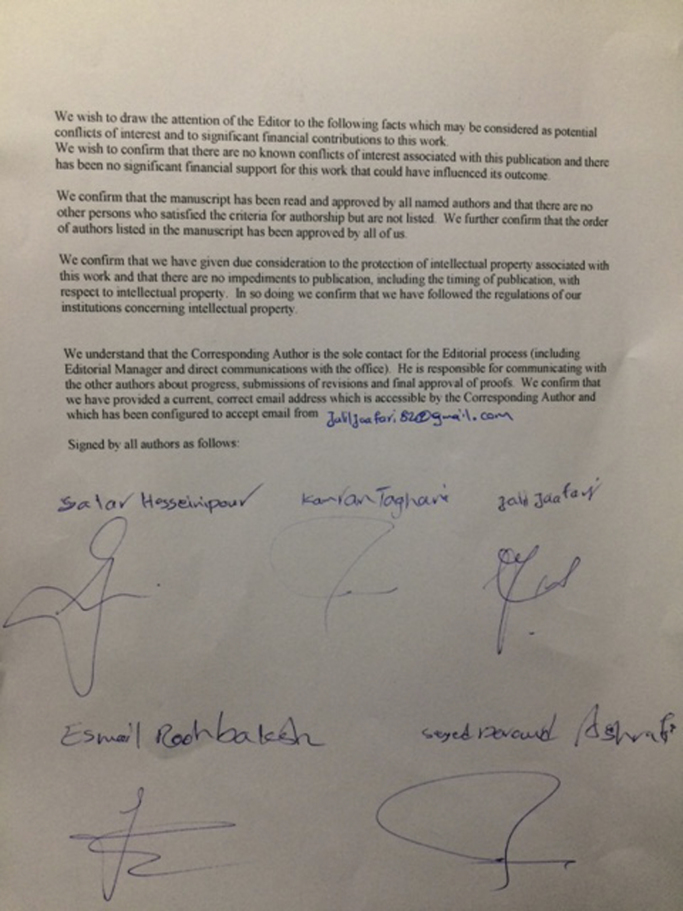# Data on pollutants content in the influent and effluent from wastewater treatment plant of Rasht in Guilan Province, Iran

**DOI:** 10.1016/j.dib.2017.11.042

**Published:** 2017-11-15

**Authors:** Salar Hosseinipour Dizgah, Kamran Taghavi, Jalil Jaafari, Esmaeil Roohbakhsh, Seyed Davoud Ashrafi

**Affiliations:** a Student at School of Health, Guilan University of Medical Sciences, Rasht, Iran; b School of Health, Guilan University of Medical Sciences, Rasht, Iran; c Department of Environmental Health, School of Public Health, Tehran University of Medical Sciences, Tehran, Iran; d Research Center of Health and Environment, Guilan University of Medical Sciences, Rasht, Iran

**Keywords:** Wastewater, Influent, Effluent, Treatment, Rasht

## Abstract

Data on this paper show the concentrations of COD, BOD_5_, TSS, K^+^, Ca^2+^, Na^+^, Cl^−^, ${NO}_{3}^{-}$, ${PO}_{4}^{2+}$, Mn^2+^, Fe^2+^, Mg^2+^, Zn^2+^, Ni, Pb, Cu and Cd in the influent and effluent of wastewater, and also the nematode eggs, total and fecal coliform in effluents from wastewater treatment plant of Rasht, Guilan Province, in Iran. Measurements of pollutants in influent and effluent was measured according to standard methods (W.E. Federation and Association, A. P. H., 2005) [1]. Statistical analysis of the data was carried out using Special Package for Social Sciences (SPSS 16).

**Specifications Table**

**Table:** 

**Subject area**	Environmental Engineering
**More specific subject area**	Pollutants in effluents
**Type of data**	Figure and table
**How data was acquired**	BOD measurement was carried out with a manometer instrument
	COD, $NO_{3}^{-}$, $PO_{4}^{2+}$ measurements were carried out using a digital reactor block, and Palintest 5000 colorimeter based on standard procedures.
	TSS were measured by drying oven.
	Digital pH meter (Metrohm) was applied for pH analyzing.
	Electrochemical probes was used for DO measuring
	Metals and nonmetals measured with ICP and Flame Photometer
	Total and fecal coliform was measured with membrane filtration technique
**Data format**	Raw, analyzed
**Experimental factors**	The data were obtained in two season, summer and winter, and the pH and DO measured in the place other samples from influent and effluent in polyethylene bottles were stored in a dark place at 4 °C temperature until the analysis.
**Experimental features**	COD, BOD_5_, TSS, K^+^, Ca^2+^, Na^+^, Cl^-^, $NO_{3}^{-}$, $PO_{4}^{2+}$, Mn^2+^, Fe^2+^, Mg^2+^, Zn^2+^, Ni, Pb, Cu, Cd, total and fecal coliform and nematode eggs were determined and compared with standard
**Data source location**	Rasht, Guilan Province, Iran
**Data accessibility**	The data are available within this paper.

**Value of the data**

● The data shown here can be used for the wastewater plant managers for proper operation.
● The data will be useful for application of treated wastewater for irrigation of plants and crops or discharge in surface waters.
● The data present here will be valuable for health risk assessment of pollutants for effluent disposal.

## 1 Data

The data give information about the situation of wastewater quality in the influent and effluent of treatment plant for both season of winter and summer. In addition, it shows the removal efficiency of these parameters after treatment. The mean concentrations of COD, BOD_5_, TSS, K^+^, Ca^2+^, Na^+^, Cl^−^, $NO_{3}^{-}$, $PO_{4}^{2+}$, Mn^2+^, Fe^2+^, Mg^2+^, Zn^2+^, Ni, Pb, Cu and Cd in influent wastewater samples were 263.7, 102.1, 82.6, 33, 192, 96, 195.2, 28.7,3.4, 0.4, 0.67, 8, 0.39, 0.3, 0.108, 0.245 and 0.00153 mg/L, respectively. Although in effluent these values were 49.3, 22.7, 35.7, 25, 125, 79, 142, 18.6, 2.25, 0.3, 0.52, 7.9, 0.15, 0.0215, 0.00934, 0.119 and 0.000064 mg/L, respectively. Moreover, the nematode eggs in effluent non- detects and total and fecal coliform in effluents were 273 and 112.5 MPN/100 mL, respectively. As shown in Table 1, total mean concentrations are always higher in the influent than effluent. In Table 2, the value of removal efficiencies for COD, BOD_5_, TSS, K^+^, Ca^2+^, Na^+^, Cl^−^, $NO_{3}^{-}$, $PO_{4}^{2+}$, Mn^2+^, Fe^2+^, Mg^2+^, Zn^2+^, Ni, Pb, Cu and Cd in winter and summer are shown.

**Table 1:** Mean and standard deviation of values of COD, BOD_5_, TSS, K^+^, Ca^2+^, Na^+^, Cl^−^, $NO_{3}^{-}$, $PO_{4}^{2+}$, Mn^2+^, Fe^2+^, Mg^2+^, Zn^2+^, Ni, Pb, Cu, Cd, nematode eggs, total and fecal coliform in influent and effluent.

**Parameter**	**Units**	**Winter**		**Summer**		**Standards for discharge to surface waters**	**Standards for agricultural use**
		Influent	Effluent	Influent	Effluent		
COD	mg/L	239.7±47.1	46.1±7.3	287.8±24.7	52.6±4.1	60	200
BOD_5_	mg/L	95.5±16.6	20.5±2.4	108.8±38.7	25±6.3	30	100
pH	–	7.6±0.3	7.8±0.3	7.8±0.3	8±0.2	6.5–8.5	6.5–8.4
DO	mg/L	1±0.2	2.6±0.5	1.3±0.3	2.9±0.7	2	2
TSS	mg/L	67.7±10.1	35.3±4.9	97.5±15.5	36.1±3	40	100
EC	ds/m	0.0799±0.008	0.0721±0.008	0.1150±0.01	0.1054±0.009	–	2.97
K^+^	mg/L	34±2	26±2	32±4.5	24±3.6	–	–
Ca^2+^	mg/L	210±15	130±6.2	174±4.5	120±2.6	75	–
Na^+^	mg/L	90±6	75±2	102±4.5	83±4.3	–	–
Cl^−^	mg /l	124.2±2.1	53.2±0.9	266.2±18.5	230.7±7	600	600
$NO_{3}^{-}$	mg/L	30±7.7	18.2±4.3	27.4±6	19±4.8	50	–
$PO_{4}^{2+}$	mg/L	3.5±0.5	2.3±0.3	3.4±0.6	2.2±0.6	6	–
Mn^2+^	mg/L	0.32±0.01	0.29±0.01	0.48±0.05	0.31±0.04	1	1
Fe^2+^	mg/L	0.63±0.02	0.46±0.01	0.71±0.07	0.58±0.05	2	2
Mg^2+^	mg/L	6.8±0.1	6.7±0.1	9.3±0.8	9.2±0.8	100	100
Zn^2+^	mg/L	0.4±0.01	0.12±0.1	0.39±0.04	0.18±0.03	2	2
Ni	µg/L	29±1	22±1	31±9	21±2	2000	2000
Pb	µg/L	9.48±0.001	8.24±0.001	12.22±0.2	10.44±0.1	1000	1000
Cu	µg/L	190±10	120±10	300±45	118±20	1000	200
Cd	µg/L	0.21±0.01	0.11±0.01	2.86±0.06	1.17±0.03	100	50
Total *Coliform*	MPN/100 mL	–	289±105	–	257±88	1000	1000
Fecal *Coliform*	MPN/100 mL	–	105±24	–	120±57	400	400
Nematode eggs	Number / L	–	0	–	0	–	1>

**Table 2:** Removal efficiency of COD, BOD_5_, TSS, K^+^, Ca^2+^, Na^+^, Cl^−^, $NO_{3}^{-}$, $PO_{4}^{2+}$, Mn^2+^, Fe^2+^, Mg^2+^, Zn^2+^, Ni, Pb, Cu, Cd from wastewater treatment plant.

**Parameter**	**Removal efficiency (%)**	
	**Winter**	**Summer**
COD	80.7	81.7
BOD_5_	78.5	77
TSS	47.8	62.9
EC	9.7	8.3
K^+^	23.5	25
Ca^2+^	38.1	31
Na^+^	16.6	18.6
Cl^−^	57.1	13.3
$NO_{3}^{-}$	39.3	30.6
$PO_{4}^{2+}$	34.2	35.3
Mn^2+^	9.3	35.4
Fe^2+^	26.9	18.3
Mg^2+^	1.4	1
Zn^2+^	70	53.3
Ni	24.1	32.2
Pb	13	14.5
Cu	36.8	60.6
Cd	47.6	59.1

## 2 Experimental design, materials and methods

### 2.1 Study area description

**Fig. 1:**
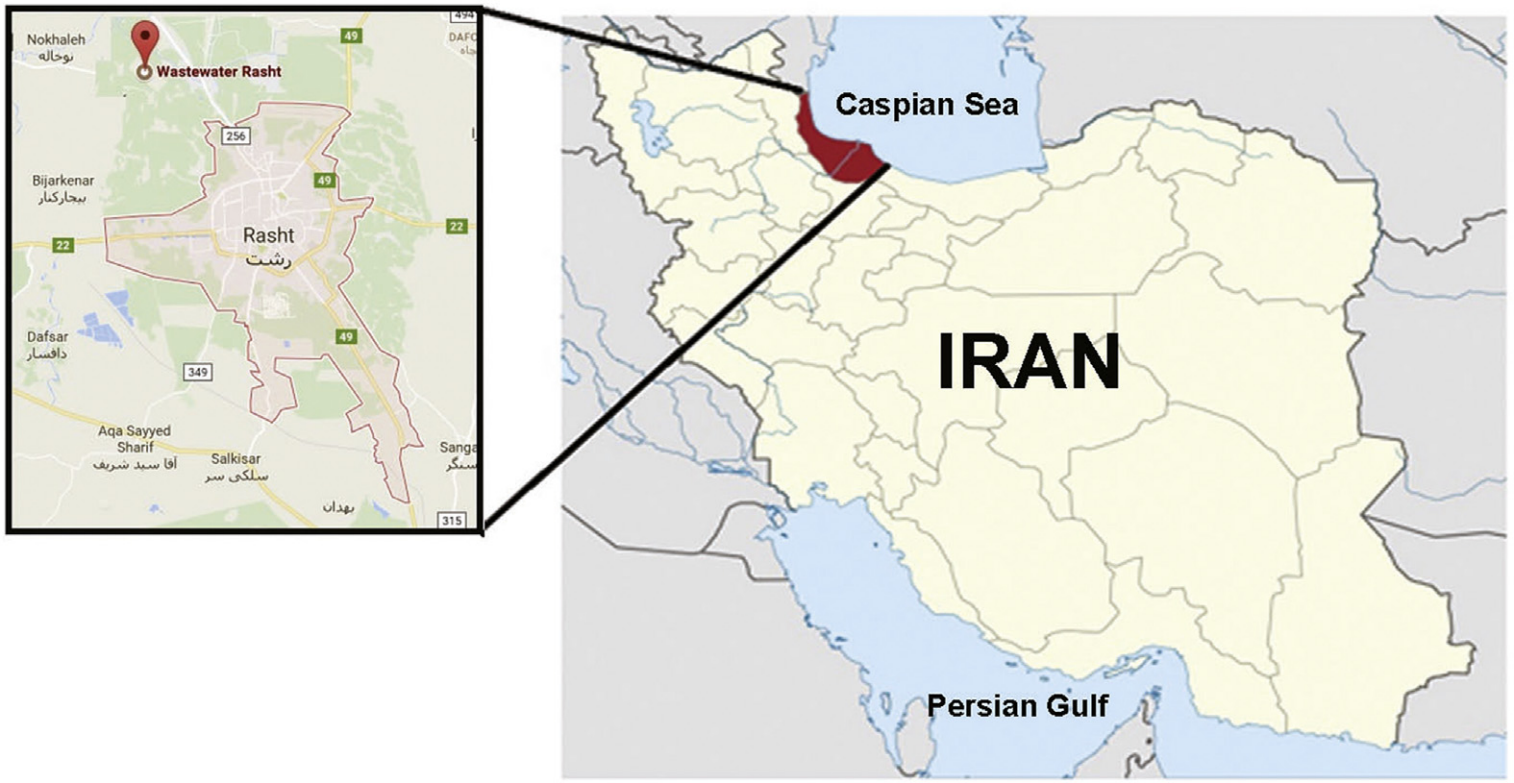
Location of wastewater treatment plant.

The selected Wastewater Treatment Plant located in Rasht city, Guilan Province, Iran, which the place of it is shows in Fig. 1. The Rasht Wastewater Treatment Plant treats more than 153,000 m^3^ of wastewater per day. It is a conventional activated sludge plant consisting of bar screen, grit chamber and the sedimentation tank and activated sludge tank and secondary settling tank. Disinfection was taken by chlorination of effluent.

### 2.2 Sample collection and analytical procedures

Experimental period was from January to February as winter and June to August as summer seasons. The 2 weekly samples were collected from both influent and effluent of wastewater treatment plant by a grab sampling method and analyzed based on standard methods for water and wastewaters for COD, BOD_5_, TSS, K^+^, Ca^2+^, Na^+^, Cl^−^, $NO_{3}^{-}$, $PO_{4}^{2+}$, Mn^2+^, Fe^2+^, Mg^2+^, Zn^2+^, Ni, Pb, Cu, Cd, nematode eggs, total and fecal coliform as an important parameters [1], [2], [3], [4]. Statistical analysis of the data was carried out using Special Package for Social Sciences (SPSS 16).

## Figures and Tables

**Fig. S1:**